# Optical monitoring of retinal respiration in real time: 670 nm light increases the redox state of mitochondria

**DOI:** 10.1016/j.exer.2016.09.006

**Published:** 2016-11

**Authors:** Pardis Kaynezhad, Ilias Tachtsidis, Glen Jeffery

**Affiliations:** a Dept of Medical Physics and Biomedical Engineering, University College London, UK; b Institute of Ophthalmology, University College London, UK

**Keywords:** Retinal imaging, Cytochrome c oxidase, Macular degeneration

## Abstract

Mitochondria play a key role in ageing and disease. Their membrane potentials and ATP production decline with age and this is associated with progressive inflammation, cell loss and death. Here we use broadband Near-Infrared Spectroscopy (NIRS) to non-invasively measure in-vivo changes in aged retinal mitochondrial respiration following exposure to 670 nm, which improves mitochondrial performance and reduces inflammation. Low power NIR light was shone into the eye via a fibre optic and the reflection monitored to measure signature changes in the oxidation of cytochrome c oxidase (COX) in complex IV of the electron transport chain. Changes in retinal haemodynamics and oxygenation were also recorded simultaneously with COX by measuring changes in oxygenated and deoxygenated haemoglobin (Δ[HbO_2_] and Δ[HHb]). Retinae of aged rats exposed to 670 nm for 5 mins showed consistent progressive increases in oxidation of COX 5 mins post exposure. This remained significantly greater than baseline for up to 2 h. This was not seen when retinae were exposed to 420 nm light of the same power or when no light was applied. 670 nm exposure significantly increased total haemoglobin concentration (Δ[HbT] = Δ[HbO_2_] +Δ[HHb]) but not haemoglobin difference (Δ[HbDiff] = Δ[HbO_2_] -Δ[HHb]). There were no changes in blood metrics in association with 420 nm light or when no light exposure was given. Hence, brief 670 nm exposure that is associated with reduced inflammation has a significant positive impact on the redox state of COX in aged retinae. The relative redox state of retinal COX may provide a valuable biomarker in ageing and macular degeneration where declining mitochondrial function is implicated.

## Highlights

• We measure real time retinal reflections in vivo in rats.
• We isolate signals from oxy/deoxy haemoglobin and those from relative COX redox.
• 670 nm increases COX redox and total haemoglobin improving mitochondrial function.
• These measurements monitor retinal respiration and may be a biomarker for disease.

## 1 Introduction

The retina has the greatest energy demand in the body because of the high metabolic rate of photoreceptors, which are rich in mitochondria (Linsenmeier and Padnick-Silver, 2000). However, a key feature of ageing is mitochondrial decline partly due to progressive mutations in their DNA (Harman, 1981, Lane, 2006, Kujoth et al., 2007). The consequence is a reduction in mitochondrial membrane potentials and ATP production in the aged retina (Kokkinopoulos et al., 2013, Calaza et al., 2015). With age there is also a progressive increase in the production of pro-inflammatory reactive oxygen species (López-otín et al., 2013). Hence, ageing is associated with a reduction cellular energy and progressive inflammation that leads to cell loss and death.

Cytochrome c oxidase (COX) in complex IV of the mitochondrial respiration chain has key points of deep red and infrared light absorbance, which can change the redox state of the copper heme in COX (Karu, 2008, Mason et al., 2014, Gonzalez-Lima and Rojas, 2011). Recently, absorbance of one of these wavelengths, 670 nm light, in the retina of aged animals has been shown to improve mitochondrial respiration, increasing their membrane potentials and improve ATP production (Kokkinopoulos et al., 2013, Gkotsi et al., 2014, Calaza et al., 2015). This in turn is associated with reduced retinal inflammation both in normal mice and those that are murine models of age related macular degeneration where inflammation appears early and is relatively aggressive (Kokkinopoulos et al., 2013, Begum et al., 2013, Catchpole et al., 2013). It has the same impact on aged drosophila, but here in this relatively short lived animal it also increases average lifespan (Begum et al., 2015) consistent with the mitochondrial theory of aging (Harman, 1981).

It has been demonstrated that it is possible non-invasively to optically image the in-vivo relative redox state of the copper heme in COX and as such, provide an indirect real time metric of mitochondrial activity using broadband near-infrared spectroscopy (NIRS). This has been reviewed by Bale (Bale et al., 2016). NIRS is a non-invasive, non-ionising technique able to measure in-vivo, real-time tissue oxygenation, haemodynamics and metabolism. It is based on the relative transparency of biological tissues to light in the near-infrared (NIR) region (650–1000 nm) and employs the fact that haemoglobin and COX express different reflective spectral elements when bound to oxygen, hence can be spectroscopically separated.

However, measuring COX in-vivo is challenging because its concentration is an order of magnitude smaller than that of haemoglobin. This issue is resolved by using broadband spectrometers and applying >100 wavelengths in the measurement algorithm, which significantly reduces error and provides accurate measurement of COX (Bale et al., 2016, Arifler et al., 2015). Measurement of oxidised-COX (oxCOX) is a reliable marker of cellular oxidative metabolism in a variety of brain measurements in both clinical (adults and neonates) and preclinical research (Bainbridge et al., 2014, Bale et al., 2014, Tisdall et al., 2007). Here we ask if it is possible to measure the relative changes in oxy and deoxy-haemoglobin as well as the oxCOX simultaneously in the retina with broadband NIR reflected light (700 nm-900 nm) where the part of the reflected spectrum originating from COX is monitored in response to 670 nm light exposure that improve mitochondrial function. We reveal that such reflected light can be used to monitor the relative redox state of retinal COX and that this may provide a valuable biomarker in ageing and AMD.

## 2 Material and methods

### 2.1 Ethical statement

All animals were used with University College London ethics committee approval and under a UK Home Office project license (PPL 70/8379). All animal procedures conformed to the United Kingdom Animal License Act (1986) and local regulations.

### 2.2 Animals

We use aged female rats (approximately 12months). Nine were used for 670 nm exposure (6 Dark Agouti and 3 Lister hooded) and 4 Lister hooded were used as controls and for 420 nm exposure. Rats have the advantage of larger eyes than found in mice. Animals were anaesthetised with 0.3 ml of a mixture of ketamine (0.56mls) and Dormitor (0.37mls) in water (0.56 ml) and their pupils dilated with Tropicamide. They were then secured on a bite bar with the body supported.

### 2.3 Retinal imaging

A single-channel portable broadband NIRS system called Mini-CYRIL (CYtochrome Research Instrument and appLication system (Kaynezhad et al., 2016) was developed to measure changes in retinal oxygenation and haemodynamics via measurement of oxygenated and deoxygenated haemoglobin; Δ[HbO2] and Δ[HHb] as well as cellular oxidative metabolism via the measurement of cytochrome c oxidase Δ[oxCOX]. The broadband NIRS system comprised a miniature white light source (HL-2000-HP), a miniature broadband spectrometer QE65pro (Ocean Optics, USA) and two optical fibres held in a micromanipulator and secured against the corneal surface which had a layer of viscotears placed on it to stop dehydration. Light from a thermally stabilised 20W halogen-tungsten bulb was filtered for the NIR region (cut-on>695 nm) and transmitted to the retina through the dilated pupil, using a 600 μm single core, 2 m long optical fibre with a numerical of aperture of 0.37 (LOPTEK, Germany). The retina was fully illuminated and the total output power transferred was <6 mW. No thermal effects or damages were observed. An identical optical fibre was used to collect the back reflected light from the retina through the detector optode securely mounted at ∼40° with respect to the source optode. The optical fibre probes held in a micromanipulator were advanced at approximately 90° to the corneal surface to avoid specular reflection from the eye. The detected light was focused onto the entrance slit (200 μm width x 1 mm height) of the spectrometer (F number: 4) with 600 gr/mm grating and 362 nm bandwidth. Real-time Spectral data between 700 and 1100 were acquired and focused on an integrated back-thinned CCD detector (Hamamatsu S7031-1006) with a sampling frequency of 0.1 Hz giving a signal of 20,000-30,000 electrons per digital conversion at 800 nm with an approximate spectral resolution of 3.15 nm. Real-time changes in retina concentration of oxygenated and deoxygenated haemoglobin (Δ[HHb], Δ[HbO_2_]) as well as oxidised Cytochrome c oxidase Δ[oxCOX] were calculated using changes in the attenuation of light between 780 and 900 nm and the UCLn algorithm (see Equation (1)), which is based on the modified Beer-Lambert law (Matcher et al., 1995).

(1) $$\left[\begin{array}{c} \Delta c_{HbO2}\\ \Delta c_{HHb}\\ \Delta c_{OxCCO} \end{array}\right]={\left[\begin{array}{ccc} \varepsilon_{HbO2}\left(\lambda_{780}\right) & \varepsilon_{HHb}\left(\lambda_{780}\right) & \varepsilon_{OxCCO}\left(\lambda_{780}\right)\\ \varepsilon_{HbO2}\left(\lambda_{781}\right) & \varepsilon_{HHb}\left(\lambda_{781}\right) & \varepsilon_{OxCCO}\left(\lambda_{781}\right)\\ \vdots & \vdots & \vdots \\ \varepsilon_{HbO2}\left(\lambda_{900}\right) & \varepsilon_{HHb}\left(\lambda_{900}\right) & \varepsilon_{OxCCO}\left(\lambda_{900}\right) \end{array}\right]}^{-1}\left[\begin{array}{c} \Delta A\left(\lambda_{780}\right)\\ \Delta A\left(\lambda_{781}\right)\\ \vdots \\ \Delta A\left(\lambda_{900}\right) \end{array}\right]\;$$

The UCLn algorithm is a least square fitting procedure to determine the chromophores concentration change, Δ*c*(*μM*·*cm*), based on the best fit between the known specific extinction coefficients, *ε*(*OD*/*μM*/*cm*) and the measured change in light attenuation, Δ*A*(Δ*OD*), over 120 wavelengths, (*λ*_780_−*λ*_900_(*nm*)). For information about specific extinction coefficients please visit UCL medical physics website (BORL, 2005).

Changes in haemoglobin difference (Δ[HbDiff] = Δ[HbO_2_] – Δ[HHb]) as a measure for retinal blood oxygenation and changes in total haemoglobin (Δ[HbT] = Δ[HbO_2_] + Δ[HHb]) indicative of retinal blood volume, were also derived. All the concentration values over the course of each experiment indicated the change with respect to the first measurement.

**Fig. 1:**
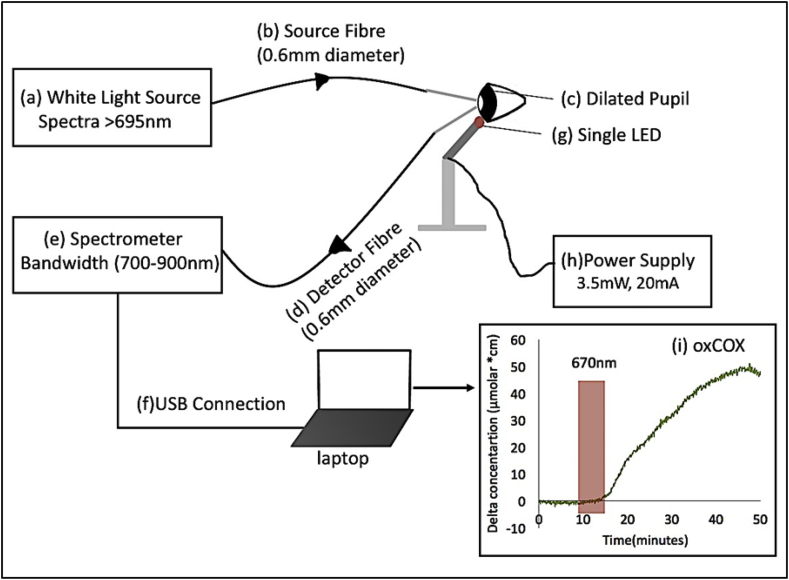
Experimental setup; Light from a thermally stabilised halogen-tungsten white light source (a) is filtered for spectra longer than 695 nm and via a 0.6 mm diameter optical fibre (Numerical aperture 0.37) illuminates the retina through the fully dilated pupil (c). The back reflected light is collected via an identical optical fibre (d) and enters the optical bench of a miniature spectrometer (e) with 700–900 nm bandwidth, through a 200 μm wide slit. The spectral data are sent to the laptop via USB connection (f). Changes in the attenuation of back reflected light are converted to real-time changes in the concentration of oxy and deoxy haemoglobin ([HbO2] and [HHb]) as well oxidised cytochrome c oxidase (oxCOX). A single 670 nm LED (g) is securely mounted adjacent to the optical fibres in clear line of sight of the eye approximately 5–8 mm from the cornea and fully illuminates the eye with 3.5 mW power at 20 mA (h). [oxCOX] in the retina increases following a 5 min exposure to the 670 nm LED over approximately 1 h as shown by the green line (i). (For interpretation of the references to colour in this figure legend, the reader is referred to the web version of this article.)

A single 670 nm LED (3.5 mW, 20 mA) secured on a metal rod was placed adjacent to the fibre optic probes in clear line of sight of the eye approximately 5–8 mm from the cornea. During this time rats were in a darkened environment of approximately 2lux. The eye was exposed to the 670 nm light source for 5 mins and reflections recorded over the next 1–2 h. In 4 other rats a long baseline measurement was taken without exposure to 670 nm and then the eye was exposed to a single 420 nm blue LED at the same energy and over the same time as the 670 nm source. Light of such shorter wavelengths inhibits mitochondrial enzyme activity by reducing oxygen consumption and oxidative phosphorylation (Chen et al., 1992, Pautler et al., 1990, Rapp et al., 1990), and as such has the opposite effects of 670 nm light. Data were collected in three groups: red LED exposure, no exposure and blue LED exposure. Changes in [oxCOX], [HbT] and [HbDiff] were averaged over a 5 min window at baseline and progressively 5 min intervals after LED exposure and for the non-exposure group at 20 min after the start of measurement. The overall configuration of the experimental setup is shown in Fig. 1.

A non-parametric Kruskal-Wallis test was used to determine overall significance over the time period examined in each group. Then a Non parametric Wilcoxon rank sum test was performed on the progressive measurements of Δ[oxCOX], Δ[HbDiff], and Δ[HbT] to investigate significant changes in concentrations before and at subsequent periods after the LED exposure.

## 3 Results

**Fig. 2:**
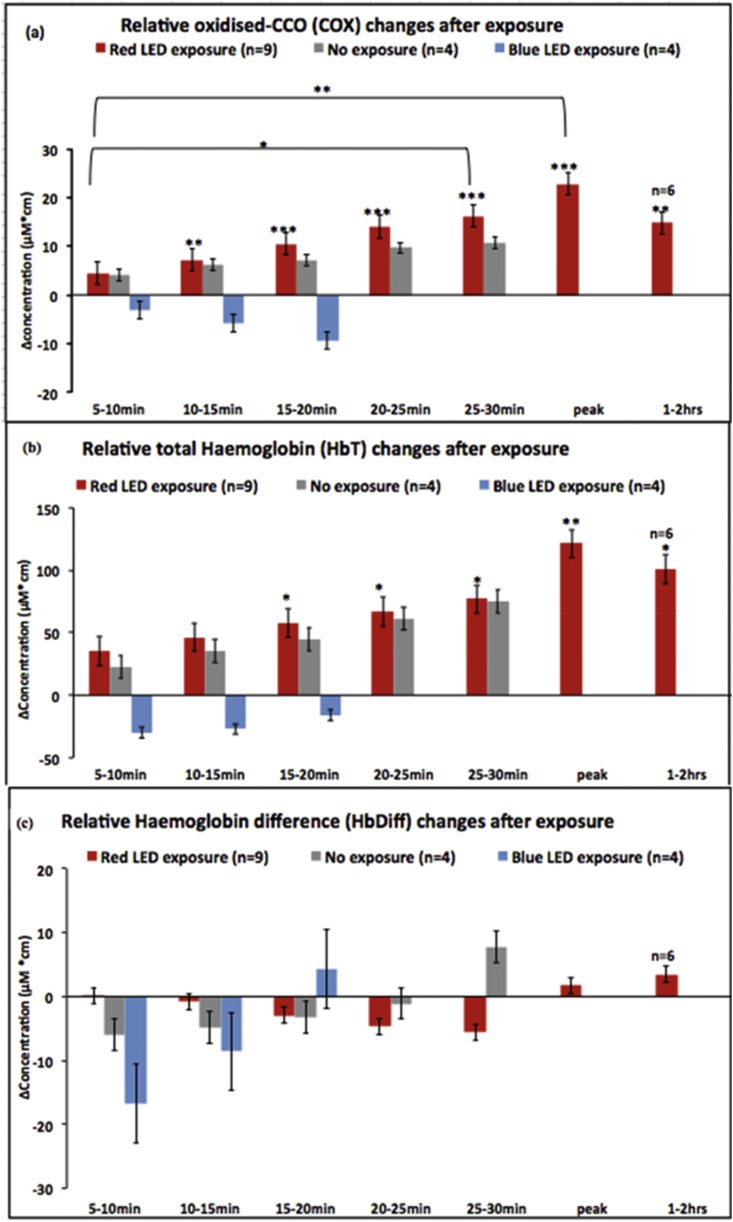
Progressive measurements following LED/hypothetical LED exposure for Δ[oxCOX], Δ[HbT] = Δ[HHb]+ Δ[HbO2] and Δ HbDiff = Δ [HbO2]- Δ [HHb], starting at 5 min after LED is off. Data is averaged over 5 min and re-baselined. Point 0 represent the time after LED exposure. Asterisks on the individual bars represent significant difference compared to baseline being zero in the graph (*p < 0.05, **p < 0.01, ***p < 0.0005). (a) There is a significant increase in retinal Δ[oxCOX] following the red LED exposure relative to baseline as early as 10 min after the LED went off (n = 9). The Δ[oxCOX] remains significantly higher for about 2 h post red LED (n = 6). There is no significant difference between the mean change in Δ[oxCOX] before and after the blue or with no exposure (n = 4). (b) Retinal total haemoglobin change (Δ[HbT], indicative of blood volume) increase significantly as early as 15 min after the red LED exposure and remains significantly higher than baseline for up to 2 h. There is no significant different between the change in [HbT] before and after blue LED exposure or when the retina is not exposed to any LED. (c) There is no significant difference in retinal haemoglobin difference (Δ[HbDiff], indicative of blood oxygenation) before and after red/blue/LED or no exposure. (For interpretation of the references to colour in this figure legend, the reader is referred to the web version of this article.)

There was a progressive increase in the [oxCOX] following the 5 mins of 670 nm exposure over baseline measurements, which is shown as the red bars in Fig. 2a. Progressive changes above baseline were first apparent as early as 5–10 mins post exposure and continued over the period of exposure (1–2 h). These changes were significant overall (p = 0.008, n = 9). This was not seen when the retina was exposed to a blue 420 nm LED (blue bars) with the same illumination power or when continuous measurement was performed without being exposed to additional LED illumination as shown with the grey bars (p = 0.9 and p = 0.7 respectively, n = 4). Changes in response to the 420 nm LED resulted in a non-significant decline in [oxCOX]. There was only a small positive drift in [oxCOX] when no light was present.

It was possible to extend the monitoring period in some animals up to 2 h and in these animals we observed that the increase in [oxCOX] reached a maximum at approximately 1 h. At this point it was >5 times greater than at 5–10 mins post exposure. The progressive increases in [oxCOX] were all statistically significant compared to baseline measurements except those taken at 5–10 mins (See Fig. 2 legend. At 1 h p = 0.00004, n = 9). Measurements at 25–30 mins and peak were also significant against those at 5–10 mins. After approximately 1 h, [oxCOX] declined gradually but was still significantly greater than baseline (p = 0.001, n = 6). It was not possible to extend the monitoring period significantly beyond 2 h in most animals because of the combined influences of age and general anaesthesia, which resulted in physiological decline that would have likely impacted upon our results.

There was a significant rise in total haemoglobin concentration ([HbT]) in the retina following 670 nm exposure reflecting increased blood volume over the monitoring period (Fig. 2b. p = 0.04, n = 9). This progressive increase was statistically significant at each stage over baseline from 15 to 20 mins post 670 nm exposure onwards. The progressive increase continued until approximately 1 h when the difference over baseline was maximal (p = 0.006, n = 9), but even after this declined, at 1–2 h it was still significantly greater than at baseline (p = 0.04, n = 6). These changes were not seen when the retina was exposed to 420 nm or left unexposed (p = 0.9 and p = 0.2 respectively, n = 4 in each group). As such the results for [HbT] reflect those found for [oxCOX] but with around a 5 min delay in terms of the progression of changes.

No significant changes were observed in the Δ[HbDiff] signal, indicative of blood oxygenation, following exposure to red or blue light. Nor was there any change in the retina when not exposed to any light (Fig. 2c).

## 4 Discussion

Our results show that exposure to 670 nm light for 5 min has a significant positive impact on the redox state of COX in the retina of old rats. This likely occurs due to the absorption of this wavelength in mitochondrial respiration. The time course of this event fits within other longer term changes that occur in response to this light. Recently we have found that following similar 670 nm light exposures, there were significant improvements in retinal mitochondrial membrane potentials and ATP production, and also reduced retinal inflammation. These were established within 3–5 days of light exposure (Kokkinopoulos et al., 2013, Gkotsi et al., 2014). Our data also showed a significant increase in retinal [HbT] although not in [HbDiff]. That we found a reduction in [oxCOX] following exposure to 420 nm light, albeit that this was not significant, is consistent with data that shows that shorter wavelengths of light suppress retinal metabolism (Chen et al., 1992, Pautler et al., 1990, Rapp et al., 1990).

While our results are clear, we cannot explicitly exclude the possibility that the light used in the analysis of reflection from the retina, which had a cut on >695 nm, was without influence. However, there was almost no overlap in the spectrum of this and the 670 nm LED. Further, there was a large difference in the energies of the two light sources. Hence, we consider that any influence was minimal and would not impact on the interpretation of our results.

The delayed increase in total haemoglobin concentration (HbT) following the 670 nm exposure and the rise in oxidised COX are indicative of an increase in retinal blood volume due to higher consumption and accordingly higher blood demand, but also possibly vasodilation. However, it is important to point out that our signal is the product of both venous and arterial flow derived with a bias towards the central retina. Further, we have no control for the possibility of venous congestion and blood volume accumulation independent of flow. For these reasons we are conservative in any possible interpretation of these results.

Mitochondria play a key role in ageing and disease as they provide the energy for cellular function. When this is reduced, inflammation becomes established and this can progress to cell death (Nunnari and Suomalainen, 2012). Here the retina is unique in two respects. First, photoreceptors have the greatest energy demand in the body (Linsenmeier and Padnick-Silver, 2000) and suffer from significant age related attrition with a 30% loss over normal life in man and rodents (Curcio, 2001, Cunea and Jeffery, 2007). Second, there are significant changes in outer retinal mitochondrial dynamics and reduced retinal ATP production that precede this cells loss. Retinal ATP declines by approximately 30% in the first 4 months of life in the mouse (Gkotsi et al., 2014) and by 12 months of age, which is before the main period of rod loss in mice, mitochondrial dynamics are markedly up regulated in photoreceptors but not in other retinal regions (Hoh Kam and Jeffery, 2015).

When inflammation becomes established or there is immune vulnerability, ageing can tip over into disease, particularly AMD, which is the largest cause of blindness in the Western world in those over 65 years (Coleman et al., 2008; Chakravarthy et al., 2010). In a mouse model of AMD, ATP production declines significantly earlier than in age matched controls. This decline is established long before the retinal phenotype develops in this model. This implies that mitochondrial function may have a role to play in the early development of AMD (Calaza et al., 2015). If ATP declines prematurely it may be reflected by changes in COX and its response to 670 nm light. Our current experiments are showing that 670 nm exposure has little impact on young animals, presumably because their mitochondria are healthy and their membrane potential do not need recharging. However, this will not be the case with age or potentially with early AMD. In such respects our findings may be of significance. In this study we directed the NIR light into the eye and assumed that the detected back reflected light was attenuated by the retina tissue and its cellular contents. The methods we have used to monitor mitochondrial function and haemodynamics are relatively simple and there is no theoretical reason why they could not be developed to evaluate age related changes in the human macular with exposure to 670 nm light, although further computational simulations of light diffusion through the eye may be needed to refine the technique for the larger eye size and to confirm the exact origin of the signal.

Currently we have no simple physiological metric based on retinal mitochondrial integrity to indicate declining function that may lead to AMD. As there is evidence that mitochondria are particularly vulnerable in animal models of this disease and that changes here are established long before the development of a retinal phenotype (Calaza et al., 2015), it is critical that a predictive biomarker is established and our simple reflective measurements may represent a step towards this.

## Disclosure statement

The authors declare no conflict of interest. All animals were used with University College London ethical committee approval. All animal procedures conformed to the United Kingdom Animals Scientific procedures Act 1986.
